# Supplementary material for: The stage- and sex-specific transcriptome of the human parasite Schistosoma mansoni
Source: Sci Data. 2023 Nov 7;10:775. doi: 10.1038/s41597-023-02674-2 (PMC10630280; doi:10.1038/s41597-023-02674-2)
Supplement: Supplementary file 1 — Supplementary Table 1 [file 41597_2023_2674_MOESM1_ESM.pdf]

Sample Name	Sex	Location	Internal Sample ID	Library Name	ENA Sample Accession	i7 Tag Sequence	i5 Tag Sequence	Lane 1 filename	Lane 1 ENA Run Accession	Lane 1 read count	Total filtered reads	Lane 1 uniquely mapped reads	Lane 2 filename	Lane 2 ENA Run Accession	Lane 2 Read count	Total filtered reads	Lane 2 unique mapped reads	Total read count	Total v10 PE mapped reads	Percent v10 PE mapped reads
X_Eggs_R1	Mix	Intramammalian	5837STDY9141995	NT1631279D	ERS4985650	TTACCGAC	CGAATACG	35101_2#3	ERR11178266	35,887,713	35,711,893	28,792,320	35207_2#3	ERR11178356	37,143,727	34,707,754	28,008,863	73,031,440	56,801,183	77.78
X_Eggs_R2	Mix	Intramammalian	5837STDY9141996	NT1631280T	ERS4985649	TCGTCTGA	GTCCTTGA	35101_2#13	ERR11178276	29,267,011	27,926,030	23,410,952	35207_2#13	ERR11178366	30,482,693	27,197,392	22,836,299	59,749,704	46,247,251	77.40
X_Eggs_R3	Mix	Intramammalian	5837STDY9141997	NT1631281U	ERS4985652	TTCCAGGT	CAGTGCTT	35101_2#23	ERR11178286	38,431,463	38,662,920	31,139,740	35207_2#23	ERR11178376	39,962,345	37,370,199	30,121,675	78,393,808	61,261,415	78.15
X_Eggs_R4	Mix	Intramammalian	5837STDY9141998	NT1631282V	ERS4985654	TACGGTCT	TCCATTGC	35101_2#32	ERR11178295	38,386,940	37,603,277	31,092,926	35207_2#32	ERR11178385	40,013,153	36,421,944	30,118,014	78,400,093	61,210,940	78.08
X_Eggs_R5	Mix	Intramammalian	5837STDY9141999	NT1631283W	ERS4985656	AAGACCGT	GTCGATTG	35101_2#41	ERR11178304	33,520,934	33,370,668	27,676,614	35207_2#41	ERR11178394	34,292,942	32,800,293	27,223,668	67,813,876	54,900,282	80.96
X_Miracidia_R1	Mix	Free-living, water	5837STDY9142000	NT1631284A	ERS4985658	CAGGTTCA	ATAACGCC	35101_2#50	ERR11178313	11,810,920	11,081,039	9,238,789	35207_2#50	ERR11178403	12,515,180	10,607,443	8,857,137	24,326,100	18,095,926	74.39
X_Miracidia_R2	Mix	Free-living, water	5837STDY9142001	NT1631285B	ERS4985660	TAGGAGCT	GCCTTAAC	35101_2#59	ERR11178322	91,165,702	89,868,791	77,502,575	35207_2#59	ERR11178412	92,175,872	89,282,000	77,003,851	183,341,574	154,506,426	84.27
X_Miracidia_R3	Mix	Free-living, water	5837STDY9142002	NT1631286C	ERS4985661	TACTCCAG	GGTATAGG	35101_2#67	ERR11178330	25,106,884	12,022,876	9,367,104	35207_2#67	ERR11178420	28,922,717	11,428,849	8,972,765	54,029,601	18,339,869	33.94
X_Miracidia_R4	Mix	Free-living, water	5837STDY9142003	NT1631287D	ERS4985664	AGTGACCT	TCTAGGAG	35101_2#5	ERR11178268	31,286,811	31,008,863	26,656,759	35207_2#5	ERR11178358	32,146,173	30,361,820	26,117,974	63,432,984	52,774,733	83.20
X_Miracidia_R5	Mix	Free-living, water	5837STDY9142004	NT1631288E	ERS4985665	AGCCTATC	TGCGTAAC	35101_2#15	ERR11178278	36,697,581	36,876,449	31,613,336	35207_2#15	ERR11178368	38,496,384	35,399,825	30,362,483	75,193,965	61,975,819	82.42
X_1d_Sporocysts_R1	Mix	*in vitro*	5837STDY9142005	NT1631289F	ERS4985668	TCATCTCC	CTTGCTAG	35101_2#25	ERR11178288	32,277,847	31,838,943	26,172,977	35207_2#25	ERR11178378	33,963,863	30,541,659	25,135,832	66,241,710	51,308,809	77.46
X_1d_Sporocysts_R2	Mix	*in vitro*	5837STDY9142006	NT1631290V	ERS4985670	CCAGTATC	AGCGAGAT	35101_2#34	ERR11178297	35,892,793	35,677,673	29,621,370	35207_2#34	ERR11178387	37,594,051	34,325,361	28,519,219	73,486,844	58,140,589	79.12
X_1d_Sporocysts_R3	Mix	*in vitro*	5837STDY9142007	NT1631291W	ERS4985672	TTGCGAGA	TATGGCAC	35101_2#43	ERR11178306	35,017,895	35,134,512	28,942,807	35207_2#43	ERR11178396	36,817,188	33,654,597	27,742,076	71,835,083	56,684,883	78.91
X_1d_Sporocysts_R4	Mix	*in vitro*	5837STDY9142008	NT1631292A	ERS4985674	GAACGAAG	GAATCACC	35101_2#52	ERR11178315	29,641,288	29,545,049	24,067,975	35207_2#52	ERR11178405	30,864,285	28,558,053	23,275,383	60,505,573	47,343,358	78.25
X_1d_Sporocysts_R5	Mix	*in vitro*	5837STDY9142009	NT1631293B	ERS4985676	CGAATTGC	GTAAGGTG	35101_2#1	ERR11178264	30,832,268	31,868,421	26,041,582	35207_2#1	ERR11178354	33,177,814	29,848,971	24,422,313	64,010,082	50,463,895	78.84
X_5d_Sporocysts_R1	Mix	*in vitro*	5837STDY9142010	NT1631294C	ERS4985677	GGAAGAGA	CGAGAGAA	35101_2#69	ERR11178332	27,466,726	27,362,950	22,761,139	35207_2#69	ERR11178422	28,178,124	26,804,403	22,302,697	55,644,850	45,063,836	80.98
X_5d_Sporocysts_R2	Mix	*in vitro*	5837STDY9142011	NT1631295D	ERS4985679	TCGGATTC	CGCAACTA	35101_2#7	ERR11178270	28,967,618	27,007,667	22,216,700	35207_2#7	ERR11178360	30,375,494	26,234,530	21,609,581	59,343,112	43,826,281	73.85
X_5d_Sporocysts_R3	Mix	*in vitro*	5837STDY9142012	NT1631296E	ERS4985680	CTGTACCA	CACAGACT	35101_2#17	ERR11178280	35,109,519	33,827,742	28,333,428	35207_2#17	ERR11178370	35,920,899	33,385,587	27,971,592	71,030,418	56,305,020	79.27
X_5d_Sporocysts_R4	Mix	*in vitro*	5837STDY9142013	NT1631297F	ERS4985683	GAGAGTAC	TGGAAGCA	35101_2#27	ERR11178290	36,963,589	36,157,533	30,869,844	35207_2#27	ERR11178380	38,214,917	35,270,776	30,123,650	75,178,506	60,993,494	81.13
X_5d_Sporocysts_R5	Mix	*in vitro*	5837STDY9142014	NT1631298G	ERS4985685	TCTACGCA	CAATAGCC	35101_2#36	ERR11178299	37,279,602	36,223,301	30,582,388	35207_2#36	ERR11178389	37,972,790	35,830,165	30,252,589	75,252,392	60,834,977	80.84
F_32d_Sporocysts_R1	Female	Intramolluscan	5837STDY9142015	NT1631299H	ERS4985687	GCAATTCC	CTCGAACA	35101_2#45	ERR11178308	19,916,240	19,860,827	4,503,158	35207_2#45	ERR11178398	20,647,759	19,277,995	4,383,669	40,563,999	8,886,827	21.91
F_32d_Sporocysts_R2	Female	Intramolluscan	5837STDY9142016	NT1631300G	ERS4985689	CTCAGAAG	GGCAAGTT	35101_2#54	ERR11178317	23,616,338	23,535,954	6,501,038	35207_2#54	ERR11178407	24,220,564	23,050,267	6,385,220	47,836,902	12,886,258	26.94
F_32d_Sporocysts_R3	Female	Intramolluscan	5837STDY9142017	NT1631301H	ERS4985691	GTCCTAAG	AGCTACCA	35101_2#62	ERR11178325	32,154,542	31,690,547	8,172,841	35207_2#62	ERR11178415	33,641,955	30,572,065	7,905,397	65,796,497	16,078,238	24.44
F_32d_Sporocysts_R4	Female	Intramolluscan	5837STDY9142018	NT1631302I	ERS4985693	GCGTTAGA	CAGCATAC	35101_2#71	ERR11178334	29,669,554	27,851,289	7,442,478	35207_2#71	ERR11178424	31,162,150	27,008,423	7,238,358	60,831,704	14,680,836	24.13
F_32d_Sporocysts_R5	Female	Intramolluscan	5837STDY9142019	NT1631303J	ERS4985695	CAAGGTAC	CGTATCTC	35101_2#9	ERR11178272	25,041,123	24,578,344	8,193,221	35207_2#9	ERR11178362	25,491,812	24,299,568	8,119,470	50,532,935	16,312,691	32.28
M_32d_Sporocysts_R1	Male	Intramolluscan	5837STDY9142020	NT1631304K	ERS4985697	AGACCTTG	TTACGTGC	35101_2#19	ERR11178282	20,507,747	21,046,824	5,627,026	35207_2#19	ERR11178372	21,459,171	20,172,481	5,416,731	41,966,918	11,043,757	26.32
M_32d_Sporocysts_R2	Male	Intramolluscan	5837STDY9142021	NT1631305L	ERS4985699	GTCGTTAC	AGCTAAGC	35101_2#29	ERR11178292	33,594,820	31,819,567	8,954,939	35207_2#29	ERR11178382	35,115,864	30,912,846	8,705,919	68,710,684	17,660,858	25.70
M_32d_Sporocysts_R3	Male	Intramolluscan	5837STDY9142022	NT1631306M	ERS4985701	GTAACCGA	AAGACACC	35101_2#38	ERR11178301	24,052,782	24,774,896	6,701,453	35207_2#38	ERR11178391	25,144,038	23,743,243	6,436,225	49,196,820	13,137,678	26.70
M_32d_Sporocysts_R4	Male	Intramolluscan	5837STDY9142023	NT1631307N	ERS4985704	GAATCCGT	CAACTCCA	35101_2#47	ERR11178310	52,577,531	52,982,669	9,133,060	35207_2#47	ERR11178400	53,752,992	51,944,602	8,963,302	106,330,309	18,096,362	17.02
M_32d_Sporocysts_R5	Male	Intramolluscan	5837STDY9142024	NT1631308O	ERS4985705	CATGAGCA	GATCTTGC	35101_2#56	ERR11178319	33,075,456	32,849,017	11,294,032	35207_2#56	ERR11178409	34,384,073	31,799,664	10,955,823	67,459,529	22,249,855	32.98
X_32d_Sporocysts_R1	Mix	Intramolluscan	5837STDY9142025	NT1631309P	ERS4985707	CTTAGGAC	CTTCACTG	35101_2#64	ERR11178327	52,413,949	53,349,468	17,985,300	35207_2#64	ERR11178417	54,246,010	51,686,663	17,438,802	106,659,959	35,424,102	33.21
X_32d_Sporocysts_R2	Mix	Intramolluscan	5837STDY9142026	NT1631310I	ERS4985708	ATCTGACC	CTCGACTT	35101_2#73	ERR11178336	27,571,604	27,328,402	10,035,045	35207_2#73	ERR11178426	28,284,142	26,780,391	9,855,024	55,855,746	19,890,069	35.61
X_32d_Sporocysts_R3	Mix	Intramolluscan	5837STDY9142027	NT1631311J	ERS4985711	TCCTCATG	GTACACCT	35101_2#78	ERR11178341	21,105,095	20,382,083	5,522,652	35207_2#78	ERR11178431	22,167,144	19,649,735	5,330,092	43,272,239	10,852,744	25.08
X_32d_Sporocysts_R4	Mix	Intramolluscan	5837STDY9142028	NT1631312K	ERS4985713	AGGATAGC	CCAAGGTT	35101_2#82	ERR11178345	19,987,672	21,643,912	6,507,569	35207_2#82	ERR11178435	20,797,374	20,685,494	6,228,954	40,785,046	12,736,523	31.23
X_32d_Sporocysts_R5	Mix	Intramolluscan	5837STDY9142029	NT1631313L	ERS4985715	GGAGGAAT	GAACGGTT	35101_2#79	ERR11178342	12,935,454	13,175,297	5,007,826	35207_2#79	ERR11178432	13,397,086	12,756,662	4,845,642	26,332,540	9,853,468	37.42
F_Cercariae_R1	Female	Free-living, water	5837STDY9142030	NT1631314M	ERS4985717	GACGTCAT	CCAGTTGA	35101_2#80	ERR11178343	15,787,887	14,160,908	12,052,642	35207_2#80	ERR11178433	16,220,056	14,087,751	12,016,455	32,007,943	24,069,097	75.20
F_Cercariae_R2	Female	Free-living, water	5837STDY9142031	NT1631315N	ERS4985719	CCGCTTAA	GTCATCGT	35101_2#84	ERR11178347	17,756,605	17,194,805	13,946,659	35207_2#84	ERR11178437	18,403,434	16,757,409	13,622,626	36,160,039	27,569,285	76.24
F_Cercariae_R3	Female	Free-living, water	5837STDY9142032	NT1631316O	ERS4985722	GACGAACT	CAATGCGA	35101_2#86	ERR11178349	15,360,630	14,867,063	12,388,092	35207_2#86	ERR11178439	15,825,906	14,567,299	12,153,590	31,186,536	24,541,682	78.69
F_Cercariae_R4	Female	Free-living, water	5837STDY9142033	NT1631317P	ERS4985723	TCCACGTT	GGTTGAAC	35101_2#88	ERR11178351	18,223,529	16,946,350	14,034,844	35207_2#88	ERR11178441	18,644,522	16,839,984	13,974,698	36,868,051	28,009,542	75.97
F_Cercariae_R5	Female	Free-living, water	5837STDY9142034	NT1631318Q	ERS4985727	AACCAGAG	CTTCGGTT	35101_2#90	ERR11178353	22,046,492	20,009,204	16,083,050	35207_2#90	ERR11178443	22,853,131	19,679,706	15,856,698	44,899,623	31,939,748	71.14
M_Cercariae_R1	Male	Free-living, water	5837STDY9142035	NT1631319R	ERS4985729	GTCAGTCA	CGGCATTA	35101_2#11	ERR11178274	15,123,476	12,621,823	10,481,420	35207_2#11	ERR11178364	16,147,537	12,189,372	10,147,144	31,271,013	20,628,564	65.97
M_Cercariae_R2	Male	Free-living, water	5837STDY9142036	NT1631320K	ERS4985731	CCTTCCAT	CACGCAAT	35101_2#21	ERR11178284	22,997,858	19,473,971	16,080,101	35207_2#21	ERR11178374	24,418,934	19,008,147	15,727,336	47,416,792	31,807,437	67.08
M_Cercariae_R3	Male	Free-living, water	5837STDY9142037	NT1631321L	ERS4985733	AGGAACAC	GGAATGTC	35101_2#30	ERR11178293	22,111,013	21,163,731	17,881,725	35207_2#30	ERR11178383	23,083,597	20,543,863	17,381,539	45,194,610	35,263,264	78.03
M_Cercariae_R4	Male	Free-living, water	5837STDY9142038	NT1631322M	ERS4985735	CTTACAGC	TGGTGAAG	35101_2#39	ERR11178302	27,749,338	25,192,733	20,992,854	35207_2#39	ERR11178392	29,793,050	24,038,611	20,064,889	57,542,388	41,057,743	71.35
M_Cercariae_R5	Male	Free-living, water	5837STDY9142039	NT1631323N	ERS4985737	TACCTGCA	GGACATCA	35101_2#48	ERR11178311	24,028,945	23,497,244	19,865,693	35207_2#48	ERR11178401	25,097,280	22,711,959	19,227,421	49,126,225	39,093,114	79.58
X_Cercariae_R1	Mix	Free-living, water	5837STDY9142040	NT1631324O	ERS4985739	AGACGCTA	GGTGTACA	35101_2#57	ERR11178320	30,839,479	29,309,804	22,920,893	35207_2#57	ERR11178410	31,205,940	29,140,689	22,922,607	62,045,419	45,843,500	73.89
X_Cercariae_R2	Mix	Free-living, water	5837STDY9142041	NT1631325P	ERS4985741	CAACACAG	GATAGCCA	35101_2#65	ERR11178328	21,447,923	20,400,740	16,003,015	35207_2#65	ERR11178418	22,460,049	19,771,388	15,520,605	43,907,972	31,523,620	71.79
X_Cercariae_R3	Mix	Free-living, water	5837STDY9142042	NT1631326Q	ERS4985725	GTACCACA	CCACAACA	35101_2#74	ERR11178337	25,576,960	24,825,597	19,749,246	35207_2#74	ERR11178427	26,420,267	24,259,936	19,305,925	51,997,227	39,055,171	75.11
X_Cercariae_R4	Mix	Free-living, water	5837STDY9142043	NT1631327R	ERS4985726	CGAATACG	TTACCGAC	35101_2#2	ERR11178265	25,094,629	22,206,698	16,561,221	35207_2#2	ERR11178355	27,381,310	20,917,747	15,620,455	52,475,939	32,181,676	61.33
X_Cercariae_R5	Mix	Free-living, water	5837STDY9142044	NT1631328S	ERS4985743	GTCCTTGA	TCGTCTGA	35101_2#12	ERR11178275	27,558,724	25,708,095	19,071,232	35207_2#12	ERR11178365	29,228,943	24,682,820	18,318,320	56,787,667	37,389,552	65.84
F_2d_Somules_R1	Female	*in vitro*	5837STDY9142045	NT1631329T	ERS4985745	CAGTGCTT	TTCCAGGT	35101_2#22	ERR11178285	37,217,104	30,087,711	26,168,037	35207_2#22	ERR11178375	39,335,841	29,643,158	25,832,243	76,552,945	52,000,280	67.93
F_2d_Somules_R2	Female	*in vitro*	5837STDY9142046	NT1631330M	ERS4985747	TCCATTGC	TACGGTCT	35101_2#31	ERR11178294	21,219,579	19,906,136	14,763,901	35207_2#31	ERR11178384	22,710,301	18,952,426	14,069,976	43,929,880	28,833,877	65.64
F_2d_Somules_R3	Female	*in vitro*	5837STDY9142047	NT1631331N	ERS4985749	GTCGATTG	AAGACCGT	35101_2#40	ERR11178303	21,234,685	19,417,533	16,365,076	35207_2#40	ERR11178393	23,097,477	18,263,122	15,404,488	44,332,162	31,769,564	71.66
F_2d_Somules_R4	Female	*in vitro*	5837STDY9142048	NT1631332O	ERS4985751	ATAACGCC	CAGGTTCA	35101_2#49	ERR11178312	33,135,155	23,084,782	19,823,869	35207_2#49	ERR11178402	35,463,886	22,361,646	19,460,453	68,599,041	39,284,322	57.27
F_2d_Somules_R5	Female	*in vitro*	5837STDY9142049	NT1631333P	ERS4985753	GCCTTAAC	TAGGAGCT	35101_2#58	ERR11178321	26,946,916	21,935,535	17,606,289	35207_2#58	ERR11178411	29,777,996	20,680,360	16,623,469	56,724,912	34,229,758	60.34
M_2d_Somules_R1	Male	*in vitro*	5837STDY9142050	NT1631334Q	ERS4985755	GGTATAGG	TACTCCAG	35101_2#66	ERR11178329	47,675,928	34,126,982	30,187,161	35207_2#66	ERR11178419	52,708,438	32,610,311	28,940,980	100,384,366	59,128,141	58.90
M_2d_Somules_R2	Male	*in vitro*	5837STDY9142051	NT1631335R	ERS4985757	TCTAGGAG	AGTGACCT	35101_2#4	ERR11178267	29,742,096	29,713,378	26,920,258	35207_2#4	ERR11178357	31,501,829	28,306,929	25,657,000	61,243,925	52,577,258	85.85
M_2d_Somules_R3	Male	*in vitro*	5837STDY9142052	NT1631336S	ERS4985759	TGCGTAAC	AGCCTATC	35101_2#14	ERR11178277	24,598,676	24,130,291	21,661,079	35207_2#14	ERR11178367	25,978,279	23,116,192	20,760,829	50,576,955	42,421,908	83.88
M_2d_Somules_R4	Male	*in vitro*	5837STDY9142053	NT1631337T	ERS4985761	CTTGCTAG	TCATCTCC	35101_2#24	ERR11178287	14,446,390	14,404,395	12,880,823	35207_2#24	ERR11178377	15,329,895	13,694,470	12,241,807	29,776,285	25,122,630	84.37
M_2d_Somules_R5	Male	*in vitro*	5837STDY9142054	NT1631338U	ERS4985763	AGCGAGAT	CCAGTATC	35101_2#33	ERR11178296	23,623,195	22,703,108	20,432,867	35207_2#33	ERR11178386	24,093,106	22,478,132	20,229,990	47,716,301	40,662,857	85.22
X_2d_Somules_R1	Mix	*in vitro*	5837STDY9142055	NT1631339V	ERS4985765	TATGGCAC	TTGCGAGA	35101_2#42	ERR11178305	39,081,876	37,554,727	33,344,456	35207_2#42	ERR11178395	40,661,718	36,502,728	32,422,150	79,743,594	65,766,606	82.47
X_2d_Somules_R2	Mix	*in vitro*	5837STDY9142056	NT1631340O	ERS4985766	GAATCACC	GAACGAAG	35101_2#51	ERR11178314	41,779,703	41,767,124	37,118,495	35207_2#51	ERR11178404	43,666,563	40,249,600	35,779,082	85,446,266	72,897,577	85.31
X_2d_Somules_R3	Mix	*in vitro*	5837STDY9142057	NT1631341P	ERS4985770	GTAAGGTG	CGAATTGC	35101_2#60	ERR11178323	56,749,551	58,027,334	52,046,890	35207_2#60	ERR11178413	60,015,644	55,140,052	49,471,995	116,765,195	101,518,885	86.94
X_2d_Somules_R4	Mix	*in vitro*	5837STDY9142068	NT1631352S	ERS4985787	TTACGTGC	AGACCTTG	35101_2#18	ERR11178281	22,305,485	22,113,245	19,598,230	35207_2#18	ERR11178371	23,607,071	21,119,969	18,717,132	45,912,556	38,315,362	83.45
X_2d_Somules_R5	Mix	*in vitro*	5837STDY9142069	NT1631353T	ERS4985788	AGCTAAGC	GTCGTTAC	35101_2#28	ERR11178291	14,700,276	14,402,717	12,629,923	35207_2#28	ERR11178381	15,177,272	14,068,732	12,333,592	29,877,548	24,963,515	83.55
F_26d_Juveniles_R1	Female	Intramammalian	5837STDY9142058	NT1631342Q	ERS4985792	CGAGAGAA	GGAAGAGA	35101_2#68	ERR11178331	37,617,772	37,486,481	33,544,742	35207_2#68	ERR11178421	39,296,367	36,502,210	32,334,070	76,914,139	65,878,812	85.65
F_26d_Juveniles_R2	Female	Intramammalian	5837STDY9142059	NT1631343R	ERS4985793	CGCAACTA	TCGGATTC	35101_2#6	ERR11178269	18,817,707	19,037,704	17,118,565	35207_2#6	ERR11178359	19,666,730	18,306,076	16,459,770	38,484,437	33,578,335	87.25
F_26d_Juveniles_R3	Female	Intramammalian	5837STDY9142060	NT1631344S	ERS4985771	CACAGACT	CTGTACCA	35101_2#16	ERR11178279	16,570,080	16,304,076	14,610,323	35207_2#16	ERR11178369	17,259,493	15,787,215	14,151,732	33,829,573	28,762,055	85.02
F_26d_Juveniles_R4	Female	Intramammalian	5837STDY9142061	NT1631345T	ERS4985773	TGGAAGCA	GAGAGTAC	35101_2#26	ERR11178289	10,488,945	10,426,601	9,353,583	35207_2#26	ERR11178379	10,826,459	10,158,814	9,118,832	21,315,404	18,472,415	86.66
F_26d_Juveniles_R5	Female	Intramammalian	5837STDY9142062	NT1631346U	ERS4985776	CAATAGCC	TCTACGCA	35101_2#35	ERR11178298	32,411,201	32,308,290	29,005,197	35207_2#35	ERR11178388	33,549,366	31,405,770	28,198,400	65,960,567	57,203,597	86.72
M_26d_Juveniles_R1	Male	Intramammalian	5837STDY9142063	NT1631347V	ERS4985777	CTCGAACA	GCAATTCC	35101_2#44	ERR11178307	13,434,861	13,722,260	12,312,366	35207_2#44	ERR11178397	13,998,964	13,211,220	11,852,416	27,433,825	24,164,782	88.08
M_26d_Juveniles_R2	Male	Intramammalian	5837STDY9142064	NT1631348W	ERS4985779	GGCAAGTT	CTCAGAAG	35101_2#53	ERR11178316	20,880,475	20,271,512	18,069,197	35207_2#53	ERR11178406	20,871,333	20,377,326	18,170,472	41,751,808	36,239,669	86.80
M_26d_Juveniles_R3	Male	Intramammalian	5837STDY9142065	NT1631349A	ERS4985780	AGCTACCA	GTCCTAAG	35101_2#61	ERR11178324	33,691,182	32,928,974	29,345,165	35207_2#61	ERR11178414	34,135,123	32,699,524	29,154,608	67,826,305	58,499,773	86.25
M_26d_Juveniles_R4	Male	Intramammalian	5837STDY9142066	NT1631350Q	ERS4985784	CAGCATAC	GCGTTAGA	35101_2#70	ERR11178333	29,106,042	28,585,962	25,552,867	35207_2#70	ERR11178423	29,375,432	28,451,574	25,429,012	58,481,474	50,981,879	87.18
M_26d_Juveniles_R5	Male	Intramammalian	5837STDY9142067	NT1631351R	ERS4985785	CGTATCTC	CAAGGTAC	35101_2#8	ERR11178271	31,868,656	32,102,065	28,643,522	35207_2#8	ERR11178361	33,505,258	30,760,007	27,458,468	65,373,914	56,101,990	85.82
